# Fabrication of a Double Core–Shell Particle-Based Magnetic Nanocomposite for Effective Adsorption-Controlled Release of Drugs

**DOI:** 10.3390/polym14132681

**Published:** 2022-06-30

**Authors:** Manzoor Hussain, Touseef Rehan, Khang Wen Goh, Sayyed Ibrahim Shah, Abbas Khan, Long Chiau Ming, Nasrullah Shah

**Affiliations:** 1 Department of Chemistry, Abdul Wali Khan University Mardan, Mardan 23200, Pakistan; manzoor14ics@gmail.com (M.H.); abbas053@gmail.com (A.K.); 2 Department of Food and Nutrition, Shaheed Benazir Bhutto Women University, Peshawar 25000, Pakistan; touseefnasr@gmail.com; 3 Faculty of Data Science and Information Technologies, INTI International University, Nilai 78100, Malaysia; khangwen.goh@newinti.edu.my; 4 Department of Pharmacy, Abdul Wali Khan University Mardan, Mardan 23200, Pakistan; 5 PAP Rashidah Sa’adatul Bolkiah Institute of Health Sciences, Universiti Brunei Darussalam, Gadong BE1410, Brunei

**Keywords:** magnetic core–shell particles, composite material, thermogravimteric analysis, controlled release, adsorption kinetic model

## Abstract

There has been very limited work on the control loading and release of the drugs aprepitant and sofosbuvir. These drugs need a significant material for the control of their loading and release phenomenon that can supply the drug at its target site. Magnetic nanoparticles have characteristics that enable them to be applied in biomedical fields and, more specifically, as a drug delivery system when they are incorporated with a biocompatible polymer. The coating with magnetic nanoparticles is performed to increase efficiency and reduce side effects. In this regard, attempts are made to search for suitable materials retaining biocompatibility and magnetic behavior. In the present study, silica-coated iron oxide nanoparticles were incorporated with core–shell particles made of poly(2-acrylamido-2-methylpropane sulfonic acid)@butyl methacrylate to produce a magnetic composite material (MCM-PA@B) through the free radical polymerization method. The as-prepared composite materials were characterized through Fourier-transform infrared (FTIR)spectroscopy, scanning electron microscopy (SEM), X-ray diffraction analysis (XRD), energy-dispersive X-Ray Analysis (EDX), and thermogravimetric analysis (TGA), and were further investigated for the loading and release of the drugs aprepitant and sofosbuvir. The maximum loading capacity of 305.76 mg/g for aprepitant and 307 mg/g for sofosbuvir was obtained at pH 4. Various adsorption kinetic models and isotherms were applied on the loading of both drugs. From all of the results obtained, it was found that MCM-PA@B can retain the drug for more than 24 h and release it slowly, due to which it can be applied for the controlled loading and targeted release of the drugs.

## 1. Introduction

Magnetic nanoparticles have recently been synthesized and applied in a large number of fields, including loading and controlled release of drugs, separation of cells, enhancing magnetic resonance imaging, the treatment of cancer, and many more [1]. Nanoparticles based on iron oxide are chemically stable, easily diffusible, less toxic, and biocompatible, due to which they are highly significant [2]. The use of bare iron oxide nanoparticles has problems of low accumulation, low separation yield by magnets, and the possibility that their atoms may oxidize, decreasing the properties of dispersion and magnetism [3]. The property of having a high surface-to-volume ratio of iron oxide nanoparticles provides room for the adsorption of plasma proteins, followed by the activation of clearance mechanisms before they reach the target site. The time for the circulation of nanoparticles and decrease in the risk of adsorption of plasma proteins is also enhanced by the modification of the surface with some biocompatible polymers with a hydrophilic nature [4,5]. Iron oxide nanoparticles are mostly encapsulated by the silica, because it is inert and has ease of functionalization, and after encapsulation these nanoparticles become water-compatible [6,7]. The use of bare magnetic nanoparticles is usually prohibited for the application of drug delivery, because of the compromised biocompatibility and they do not possess various functionalities facilitating the coating of polymers on their surfaces. For this reason, they are coated with silica so that their surfaces are ready for coating and enhanced interaction with drugs [8,9,10].

Aprepitant is an orally active neurokinin receptor antagonist used for chemotherapy-induced nausea and vomiting (CINV) [11]. It is the first FDA-approved drug that is used to treat CINV that works for more than 24 h after chemotherapy treatment [12]. It has a very low affinity for serotonin (5-HT_3_), corticosteroid, and dopamine receptors. Various drugs have been developed to prevent acute and delayed emesis, but aprepitant has been approved for PONV and nausea treatment during cancer therapy [13]. Sofosbuvir is a drug that is used in combination with other drugs in order to treat hepatitis C [14]. Among the list of essential drugs, it has been included in basic health systems by the World Health Organization. By combining it with the NS5A inhibitor ledipasvir, sofosbuvir is used to treat genotypes 1, 2, 3, 4, 5, and 6, as well as hepatitis C infections [15,16,17]. Aprepitant and sofosbuvir have little water solubility, resulting in erratic bioavailability. This is because the active substance present in the drugs should be in dissolved form in aqueous form in the gastrointestinal tract, such that it crosses the luminal wall to make its therapeutic effects more significant. Moreover, higher solubility allows the active form of the drug to easily enter the blood and reach its target site to show its therapeutic effects. The loading and release of these drugs into the carrier molecules is also of great concern due to limited data available for them.

Copolymers of 2-acrylamido-2-methyl propane sulfonic acid (AMPS) with other polymers have been synthesized and applied for several purposes. Molecularly imprinted polymer of N-maleoyl chitosan with AMPS have been prepared and applied for the recognition and delivery of bovine serum albumin [18]. A copolymer of AMPS with acrylamide has been applied for adsorption of cadmium from aqueous solution and for the loading and release of 5- fluorouracil, as well as the antiviral drug indinavir sulfate [19,20,21]. Another copolymer of AMPS with polyvinyl alcohol and acrylamide has been applied for the controlled loading and release of ibuprofen [22]. Hydrogel prepared from chitosan and poly(AMPS) has been applied for the removal of methylene blue, acid red dye, Cd(II), and Cr(II) from wastewater [23]. Hydrogel prepared from poly(AMPS) and pectine has been applied for the controlled loading and release of captopril [24]. Different types of nanomaterials prepared from poly(α-L-glutamic acid) have been prepared and applied for the loading and controlled release of various types of drugs [25]. In one of the recent studies, hollow polymeric nanosphere (HPN)-supported imidazolium-based ionic liquids were shown to be efficient materials against multidrug-resistant bacteria, due to which they can also be applied in the biomedical field [26].

The fabrication of magnetic core–shell composites for drug loading and controlled release is of great value due to their efficiency and ability of controlled targeted drug delivery. The controlled loading and release of curcumin has also been demonstrated by the application of core–shell particles comprising chitosan with magnetic iron oxide nanoparticles (IONPs) and gold nanoparticles [27]. The core-shell structure made of IONPs@Au has been applied as an anticancer agent [28]. Iron oxide magnetic nanoparticles have been combined with large number of polymers for vast applications, e.g.,polylactide-co-glycolide combined with iron oxide nanoparticles has been applied for the encapsulation of both hydrophilic and hydrophobic drugs [29]. AMPS incorporated with pectin followed by the loading of IONPs has been applied for the controlled release of diclofenac sodium [30]. Poly-(sodium-2-acrylamido-2-methyl propane sulfonic acid-co-styrene)/magnetite have been used for the purpose to inhibit corrosion from steel [31].

Generally, there is a large number of drug delivery systems used, including liposomes, graphene, gold, carbon nanotubes, and magnetic nanoparticles [32,33,34,35,36]. These aforementioned materials have little ability to deliver the drug in a controlled way. Bare magnetic nanoparticles have limitations to their application, due to which they are incorporated in some polymeric materials to enhance their applicability. Current research is focused on site-specific drug delivery systems, by releasing the drug in response to an external trigger, i.e., physiological pH and temperature [37,38,39]. In order to overcome the problem of controlled drug delivery, we here focus on the synthesis of a novel drug delivery system based on the combination of magnetic nanoparticles (silica-coated IONPs) and core–shell particles (poly(2-acrylamido-2-methylpropane sulfonic acid)@butyl methacrylate). The as-prepared magnetic composite material (MCM-PA@B) was thoroughly characterized by various analytical techniques, and was applied for loading and controlled release using aprepitant and sofosbuvir as model drugs.

## 2. Materials and Methods

### 2.1. Materials

Sodium hydride 60%, magnesium turnings, and potassium persulfate were purchased from Daejung, S. Korea. Dimethylformamide (DMF), 4-vinylbenzyl chloride (VBC), 2-acrylamido-2-methylpropane sulfonic acid (AMPS), 4,4′-azobis(4-cyanovaleric acid) (ABCA), dimethyl sulfoxide (DMSO), butyl methacrylate (BMA), bromobenzene, and iodine were purchased from Sigma-Aldrich, Schnelldorf, Germany. Pyrroles and carbon disulfide were purchased from Daejung, Korea, diethyl ether from Sigma-Aldrich, Schnelldorf, Germany, magnesium sulfate and petroleum ether were from Fisher Scientific, Chicago, IL, USA, and tetrahydrofuran (THF) was purchased from Daejung, Korea. Silica-gel, FeCl_2_.4H_2_O, and FeCl_3_.6H_2_O were purchased from UniChem USA. n-Hexane was purchased from Sigma-Aldrich, Germany. Aprepitant and sofosbuvir were donated by Ferozsons pharmaceutical company, Nowshera, Pakistan.

### 2.2. Methods

#### 2.2.1. Preparation of Poly(2-acrylamido-2-methylpropane sulfonic acid) (poly(AMPS))

First of all, the RAFT agent 4-vinylbenzyl pyrrole carbodithioate (VP) was synthesized as reported in our previous study [40]. Briefly, 160 mL of DMF was taken, in which sodium hydride (6.03 g) was dissolved to produce a grey-colored suspension. Pyrrole solution was prepared by dissolving 10.02 g in DMF to make 20 mL of solution, which was further added to the mixture, forming a yellow-colored product. The temperature of this mixture was dropped to 0 °C, and CS_2_ solution already prepared from 9.01 mL in 20 mL of DMF was added to the mixture through continuous stirring for 30 min. Then, 4-VBC (22.2 g) solution in 20 mL of DMF was added with continuous stirring to produce a dark-red-colored mixture that was stirred overnight at room temperature (25 °C). This product was separated from its mixture by using a separating funnel that contained a mixture of deionized water and diethyl ether (1:1) and was further dried with 20 g of solid MgSO_4_. The product was filtered, followed by vacuum distillation, and then, using silica gel as stationary phase, the product was separated through the column. The obtained product was again vacuum-distilled, producing a yellow-colored product thatwas kept in a nitrogen-purged environment at −18 °C.

For the preparation of poly(AMPS), 1 g of the RAFT agent 4-VP was added to 25 mL of DMSO in a two-neck flask reactor. Then, 25 g of AMPS monomer was added to it, followed by the addition of 0.5 g of 4,4′-azobis(4-cyanovaleric acid) (ABCA) initiator. The flask containing the mixture was made oxygen-free by purging with N_2_ several times. Then, the mixture was heated to 60 °C for 12 h. After heating, the mixture converted to brown-colored poly(AMPS). The obtained product was stored at −5 °C in a nitrogen atmosphere, and was used further for the synthesis of core–shells. The as-prepared poly(AMPS) revealed the following NMR data:

^1^H NMR (400 MHz (D2O), ppm): 7.6 2H, (br s, N-pyrrole–H), 7.1 (br m, –Ar–), 6.3 (2H, br s, N-pyrrole–H), 3.3 (2H, br, m, C-CH2-C), 2.1 (C-CHC-C) 1.4 (6H, br m, C-CH3)

^13^C NMR (400 Mhz (D2O) ppm): 58.1 (RCH2SO3H), 52 (RCHS), 38.6 (RCH2), 26.6 (RCH3)

#### 2.2.2. Preparation of Core–Shell Latex of Poly(AMPS)@BMA (PA@B)

The fabricated Poly(AMPS) (1 g) was taken and dissolved in DI water in a reactor, and then BMA (25 mL) was added, followed by the addition of K_2_S_2_O_8_ (1 g in 15 mL of DI water). An overhead condenser was installed over the flask, and the mixture was stirred at 60 °C for 24 h in a water bath at 900 rpm using a magnetic stirrer. A milky, white-colored suspension of CS was obtained, which was transferred to a reagent bottle and stored for further use.

#### 2.2.3. Synthesis of Silica-Coated IONPs (IONPs@SiO_2_)

The synthesis of silica-coated IONPs was carried out in two consecutive steps: In the first step, a co-precipitation method was used to synthesize the magnetic IONPs, as reported by Veisi et al. [41]. Fe^2+^ and Fe^3+^ were mixed in 40 mL of double-DI water at a molar concentration of 0.15 mol and 0.3 mol, respectively. Sodium hydroxide solution (2 M) was added dropwise to keep the pH above 10, and temperature was maintained at 80 °C for 6 h. Then, the solution was cooled and filtered, followed by washing three times with distilled water. The black-colored particles were then dried in an oven to remove moisture, and then stored in an inert environment.

In the second step, the synthesized IONPs were coated with SiO_2_ using theStöber method [10]. From the synthesized IONPs, 1.6 g was taken in a flask. Then, ethanol (80 mL), deionized water (16 mL), 10% NaOH solution (16 mL), and TEOS (5 mL) were added to it, followed by sonication for 1 h at 30 °C. Blackish grey-colored particles were produced, which were further separated by using an external magnet. These synthesized silica-coated magnetic nanoparticles were further washed with distilled water and ethanol, followed by drying at 80 °C in an oven for six hours.

#### 2.2.4. Fabrication of Magnetic Composite Material (MCM-PA@B)

Next, IONPs@SiO_2_wastaken in different amounts (0.1 g to 0.5 g) and dispersed in deionized (DI) water by sonication. PA@B was added to it in different amounts (1 mL to 5 mL) and sonicated for 15 min. Then, 4,4′-azobis(4-cyanovaleric acid)(0.015 g) in 1.5 mL of DI water was added to it and again sonicated for 15 min, followed by the addition of IONPs@SiO_2_ suspension with continuous sonication for 10 min (Table 1). The as-prepared mixture was then stirred for 2 h under nitrogen-purged conditions at 60 °C toproduce the composite materials. The synthesized magnetic composite material (MCM-PA@B) was then transferred to a beaker and kept in the oven until dried and converted to a black-colored material.

#### 2.2.5. Characterization of the Synthesized MCM-PA@B

FTIR Spectrometer (Varian 640-IR, USA) was used to obtain the FTIR spectra of the prepared MCM-PA@B in the wavenumber region of 4000–400 cm^−1^. A thermogravimetric analyzer (TGA-50H Shimadzu, Kyoto, Japan) was used to study the thermal stability of the prepared composite materials. The machine was run from 25 to 600 °C ata heating rate of 30 °C/min. An X-ray diffractometer (D-2 Phraser, Bruker, Denver, CO, USA) was used to analyze the XRD patterns by applying a scan rate of 20°/min, and a Cu Kα radiation source was used. A field-emission scanning electron microscope (SEM) (model JSM5910, JEOL. Kyoto, Japan,) was used with an acceleration voltage of 30 KV to analyze the surface morphology of the composite materials. A Thermo Electron Co., USA, Helios β UV–Vis Spectrometer (Lambda 25, Perkin Elmer, Waltham, MA, USA) was used to determine the UV–Vis absorption spectra of the samples.

#### 2.2.6. Application of MCM-PA@B for Drug Loading and Release Studies

The as-prepared composite materials were further applied for the loading and release of the drugs aprepitant and sofosbuvir. A stock solution of eachdrug (100 ppm) was prepared in 100 mL of methanol through sonication at room temperature. From the stock solution, five separate sample solutions were further prepared through dilution with a concentration of 20 ppm. The composite material was added to each of the sample solutions at neutral pH and STP. All of the drug solutions containing the composite were shaken overnight. From each of the samples, aliquots were taken out after a certain interval of time and checked through the UV spectrophotometer to find the loading amount of the drug.

To optimize the conditions for maximum loading of the drugs, both of the drugs were tested for their loading at different pH and drug concentrations. For pH optimization, drug solutions were prepared, and their pH was set to2 by the addition of Britton–Robinson buffer, after which 0.05 g of composite was added. This solution was shaken for 2 h, and its absorbance was checked using the UV spectrophotometer. A similar procedure was followed for pH 4, 6, 8, and 10.

For optimization of the drug concentration used, drug solutions from 20 ppm to 100 ppm were prepared, and their pH was set to 4 by adding buffer, followed by the addition of 0.05 g of composite materials. These solutions were shaken for 2 h and then their absorbance was checked.

#### 2.2.7. Kinetic Study of Adsorption

##### Pseudo-1st-Order Kinetic Model

Drugs’ loading data were obtained and, using a pseudo-1st-order kinetic model, were used to interpret the obtained results (Equations (1) and (2)):

Log (qe − qt)= log qe − k_1_t/2.303 (1)

qe or qt = (Ci − Ce)V/m(2)

where qt is the amount of drug loaded at any time, while qe shows the amount of loaded drug at equilibrium time, k_1_ is the constant for the first order, Ce and Ci are the drug’s concentration at equilibrium and initial use, respectively, V is the volume of the drug solution, and m is the mass of the composite materials (0.1 g).

##### Pseudo-2nd-Order Kinetic Model

A second-order kinetic equation was used for the interpretation of the data (Equation (3)):

t/qt = t/qe + 1/k_2_ qe^2^ (3)

where k_2_ shows the rate constant for the second order, while qe and qt show the amount of loaded drug at equilibrium and at any time.

##### Intra-Particle Diffusion Kinetic Model

The data were also interpreted using an intra-particle diffusion model (Equation (4))

q_t =_ K_int_t½ + C (4)

where C is the intercept and is associated withthe width of the border layer i.e., the higher the value of C, the further is its boundary layer outcome.

#### 2.2.8. Equilibrium Studies

Langmuir and Freundlich isotherms were used to determine the equilibrium study of the drug incorporated into the synthesized composite materials. The Langmuir equation shows monolayer attachment of the drug with the composite materials by providing many sites for linking the drug with the composite materials. The Langmuir equation in presented in Equation (5):

Ce/qe = 1/K_L_ + a_L_ Ce/K_L_ (5)

where K_L_ shows the equilibrium adsorption constant, while qe is the monolayer loading capacity of the drug.

The Freundlich isotherm is given in Equation (6):

Log qe = log K_F_ +1/n log C_e_ (6)

The values for the constants (K_F_ and N) of Freundlich and Langmuir (Q and K_L_) as well as the regression coefficient R^2^ were calculated.

## 3. Results

### 3.1. Scanning Electron Microscope Study of MCM-PA@B

**Figure 1:**
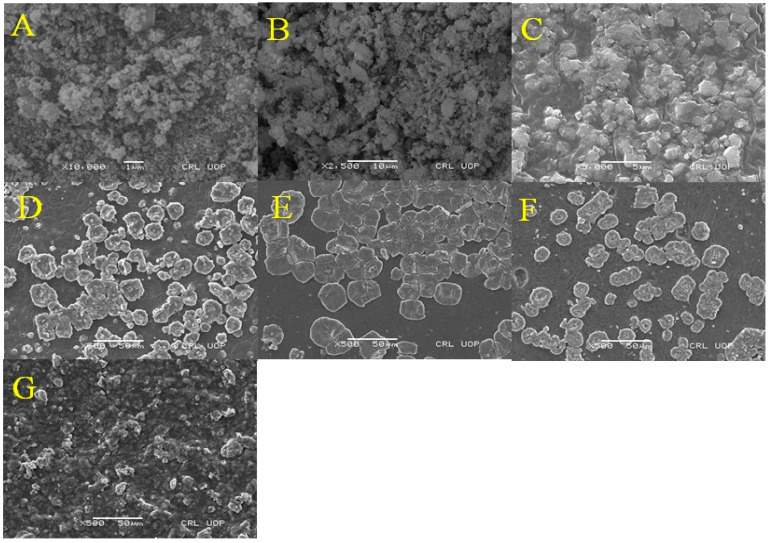
SEM images of IONPs (**A**), IONPs@SiO_2_ (**B**), MCM-PA@B (**C**–**G**), IONPs@SiO_2_1:PA@B1 (**C**), IONPs@SiO_2_1:PA@B3 (**D**), IONPs@SiO_2_1:PA@B5 (**E**), IONPs@SiO_2_3:PA@B5 (**F**), and IONPs@SiO_2_5:PA@B5 (**G**).

**Figure 2:**
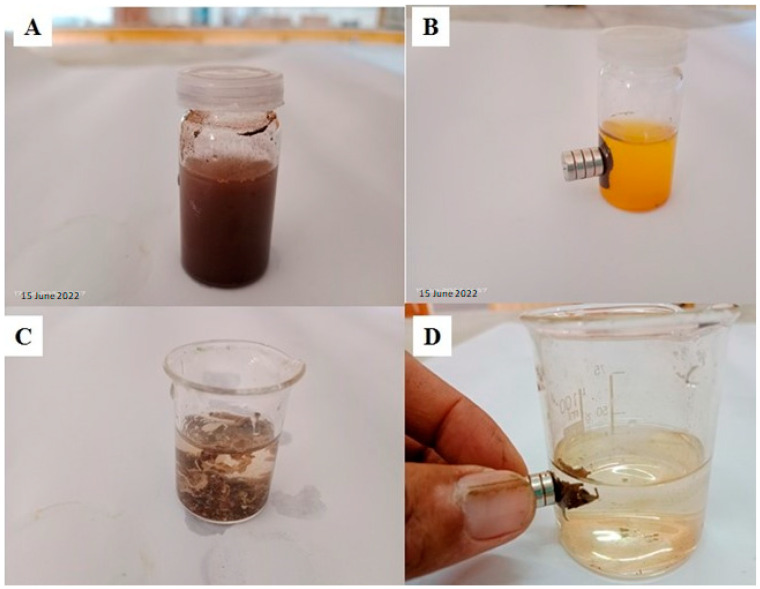
Magnetic behavior of the synthesized magnetic nanoparticles (**A**,**B**) and MCM-PA@B (**C**,**D**).

The morphological, internal, and topographical structure of the as-prepared MCM-PA@B was evaluated by SEM analysis. It can be seen in Figure 1A,B that the IONPs and IONPs@SiO_2_ had a small particle size; however, the incorporation of silica onto the IONPs converted them into rough and heterogeneous surface particles [42]. The incorporation of IONPs@SiO_2_ into the PA@B to produce MCM-PA@B can be seen in Figure 1C–G. The IONPs@SiO_2_1: PA@B1 sample contained an equal ratio of both IONPs@SiO_2_ and PA@B, due to which its morphological structure did not look uniform. However, as the IONPs@SiO_2_ were incorporated with a higher amount of PA@B, uniformity in the structure enhanced and made it more recognizable, as shown in Figure 1D–F. Moreover, the core–shell polymer of PA@B was coated over the silica-coated IONPs to form double core–shell nanocomposites. The surface polymer molecules covered the nanoparticles from all sides and, hence, prevented their agglomeration, due to which these double core–shell nanocomposites were distinguishable and clear. The similar composition of PA@B, with changes only to its quantity, did not cause significant changes in the morphology of the nanocomposites [41]. As the ratio of IONPs@SiO_2_ increased, the surface again shifted towards non-uniformity. From the SEM results, it can be seen that using the IONPs@SiO_2_ and PA@B latex at ratios of 1:3, 1:5, and 3:5 gave us composite materials with similar morphological structures. The magnetic behavior of the synthesized magnetic nanoparticles and MCM-PA@B is presented in Figure 2.

### 3.2. FTIR Study of the Composite

**Figure 3:**
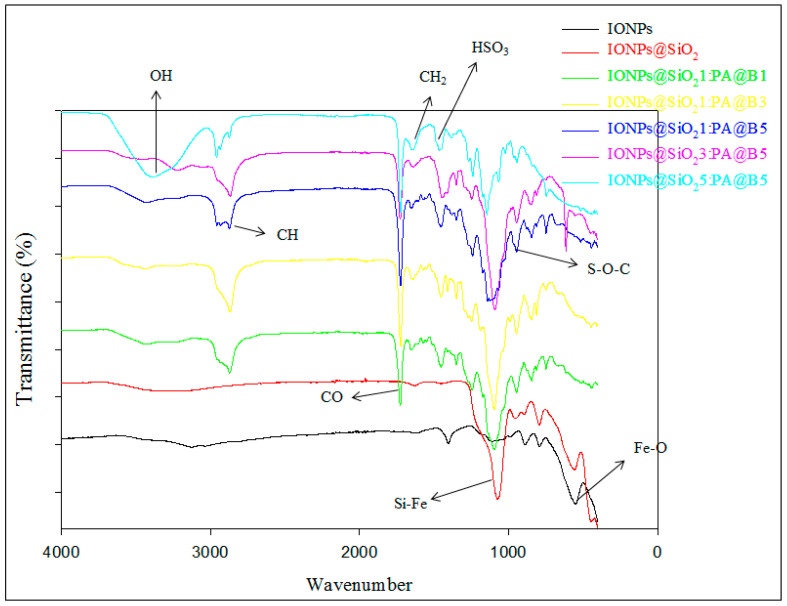
FTIR spectra of pure IONPs, IONPs@SiO_2_, and the fabricated MCM-PA@B with different ratios of IONPs@SiO_2_ and PA@B.

The synthesis of IONPs is confirmed by the presence of peak at 565 cm^−1^, which is due to the presence of stretching of Fe–O bonds in Fe_2_O_3_ in IONPs [43], while the band at 1084 confirms the silica coating on the IONPs to produce Fe_2_O_3_@SiO_2_ [44]. The bands at 1353 cm^−1^ are due to the presence of the sulfonate group of the poly(AMPS) [45].The peaks at 2872 and 1452 cm^−1^ are due to CH and CH_2_ as well as C-N stretching vibrations [46]. The broad peak at 3100–3500 cm^−1^ indicates OH stretching and the presence of the NH group. The peak at 941 cm^−1^ is due to S–O–C symmetrical stretching vibrations of the poly(AMPS) shell, while that at 1735 cm^–1^ assures the presence of a CO group, and that at 1249 cm^−1^ shows the presence of a BMA core [20] (Figure 3).

### 3.3. Thermogravimetric Analysis

**Figure 4:**
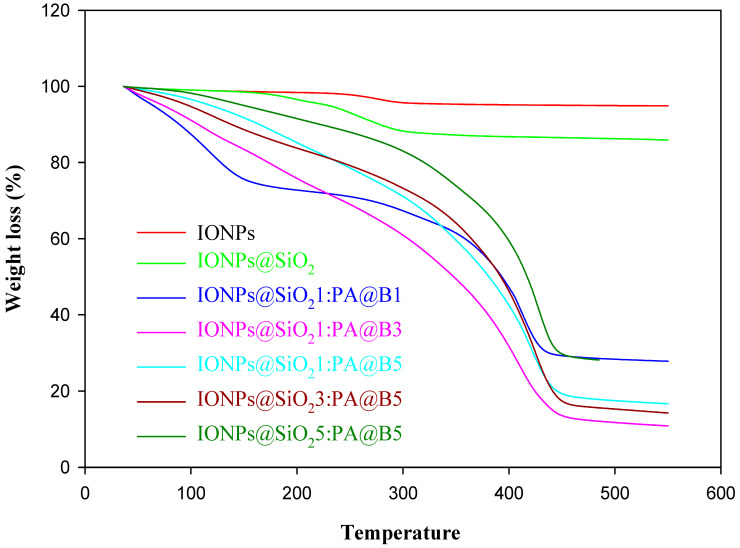
Thermogravimetric analysis of pure IONPs, IONPs@SiO_2_, and the fabricated MCM-PA@B with different ratios of IONPs@SiO_2_ and PA@B.

By looking into the spectra of TGA analysis given in Figure 4, we can observe two degradation stages for IONPs and IONPs@SiO_2_. In the first stage, the weight loss of IONPs at 125 °C is due to the loss of water molecules attached to the surface of the IONPs, while in the second stage the weight loss at 170 °C and 360 °C is due to the transformation of crystal from Fe_3_O_4_ to γ-Fe_2_O_3_ caused by heating, and only a 5% decrease in the weight of the composite occurs [47]. In the case of IONPs@SiO_2_, in the first stage, 2% weight loss occurs up to 230 °C due to the loss of any remaining water and volatile molecules, and then the weight loss decreases by 12% due to the conversion of Fe_3_O_4_ to γ-Fe_2_O_3_ at 360 °C, followed by no significant weight loss, indicating a higher degree of thermal stability [48]. All of the composites of CS and IONPs@SiO_2_ contain different amounts of poly(AMPS) and IONPs@SiO_2_. The graph shows the TGA of all of these composites, and indicates that each of the composite has three degradation stages. For the composites IONPs@SiO_2_1:PA@B1, IONPs@SiO_2_1:PA@B3, IONPs@SiO_2_1:PA@B5; the first degradation stage occurs below 150 °C, showing a 10% loss of water and volatile molecules contents [46], followed by degradation up to 300 °C, showing the loss of sulfonate groups of the polymers up to 30%, and then 85% weight loss occurs at 450 °C due to the degradation of the polymer on the IONPs@SiO_2_. Above 450 °C, there is no significant change in the weight, confirming the presence of IONPs, as these particles have high thermal stability. Moreover, for the composites IONPs@SiO_2_3:PA@B5 and IONPs@SiO_2_5:PA@B5, both have high amounts of IONPs as compared to previous composites, so they have relatively high thermal stability, and a maximum of 70% weight loss occurs at 450 °C, followed by no significant decrease in the weight of the samples. As IONPs@SiO_2_5:PA@B5 contains more IONPs@SiO_2_, it gives more thermal stability to the composite, due to which its graph is slightly above that of the IONPs@SiO_2_3:PA@B5 [31,46]. From the above discussion it can be concluded that composite materials with high concentrations of IONPs@SiO_2_ are more thermally stable than those with low amounts of IONPs@SiO_2_.

### 3.4. XRD Analysis

**Figure 5:**
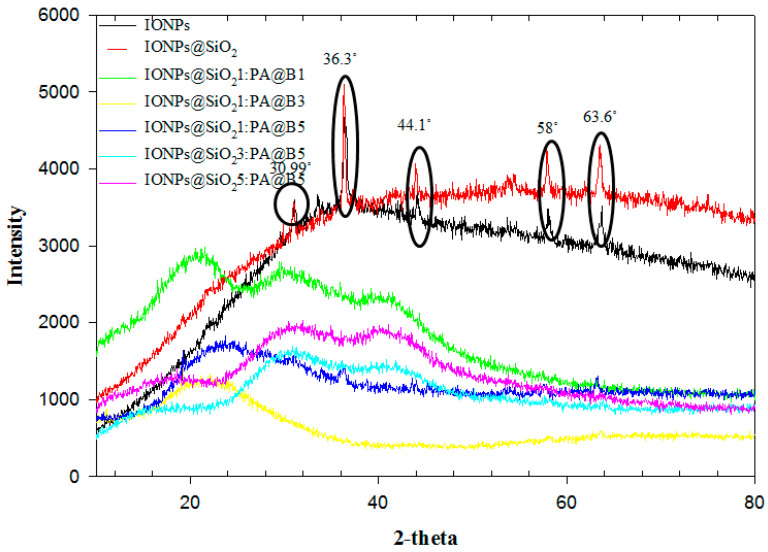
XRD spectra of pure IONPs, IONPs@SiO_2_, and the fabricated MCM-PA@B with different ratios of IONPs@SiO_2_ and PA@B.

The XRD analysis results of the synthesized composite materials are given in Figure 5. The data from the graph show us that the IONPs have some sharp peaks that confirm their crystalline structure. Moreover, the crystalline form of the nanoparticles is confirmed by the sharp peaks at 30.99°, 36.3°, 44.1°, 58°, and 63.6° [46]. The graph of the IONPs@SiO_2_ also shows similar peaks that ensures its crystalline nature, with no loss of any of the entity. This shows that after coating of of silica on the IONPs, the crystalline structure of the nanoparticles was not disturbed, and showed the peaks in the similar range. By incorporation of the core–shell latex with IONPs@SiO_2_, the intensity of these peaks decreased and became very low, ensuring their combination. However, due to the presence of the IONPs@SiO_2_, the peaks are still present with low intensity, confirming the presence of both the PA@B and IONPs@SiO_2_.

### 3.5. Zero-Point Charge (pHzpc) on the Composite

**Figure 6:**
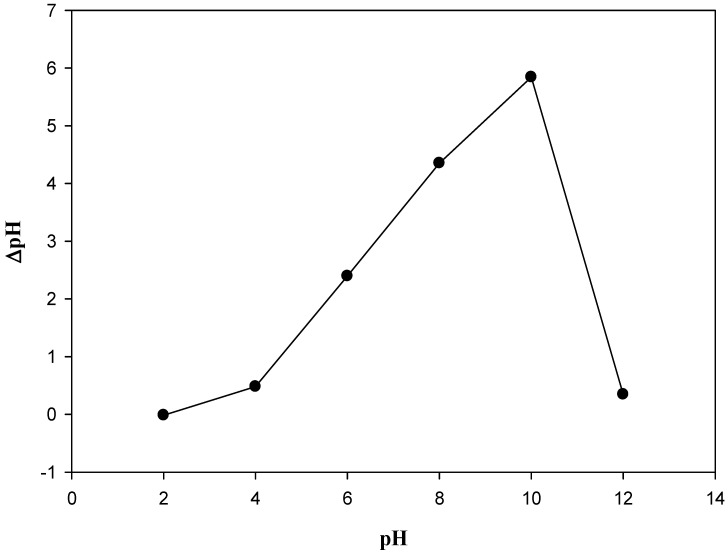
pHzpc of the fabricated composite materials (MCM-PA@B).

The PA@B contains a sulfonate group from the poly(AMPS) shell, which is incorporated with the IONPs@SiO_2_. Due to the presence of these acidic sulfonate groups, the pHzpc of the composite material was expected to be in the acidic range, as confirmed by the results obtained, with a value of 2.5. Below the pHzpc value, the composite has positive charge, while increasing above this value it has negative charge (Figure 6).

### 3.6. EDX Analysis

**Figure 7:**
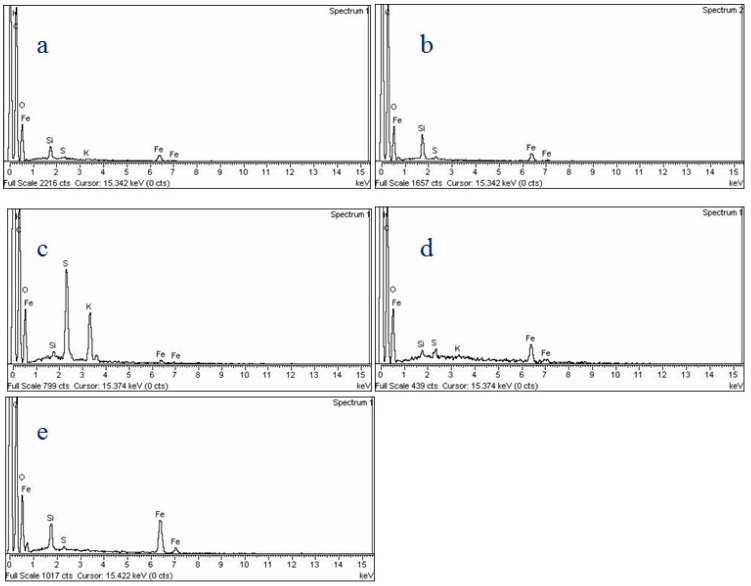
EDX analysis of the IONPs@SiO_2_1:PA@B1 (**a**), IONPs@SiO_2_1:PA@B3 (**b**), IONPs@SiO_2_1:PA@B5 (**c**), IONPs@SiO_2_3:PA@B5 (**d**), and IONPs@SiO_2_5:PA@B5 (**e**).

Figure 7 shows the EDX analysis of the synthesized IONPs. The incorporation of silica with the IONPs is confirmed by the presence of its peaks in Figure 7a. Moreover, the presence of the peaks for carbon and sulfur also shows the presence of the core–shell latex linked with IONPs@SiO_2_. The presence of peaks for Fe confirms the magnetic nature of all of the prepared MCM-PA@B (Figure 7).

### 3.7. Drug Loading and Release Study

**Figure 8:**
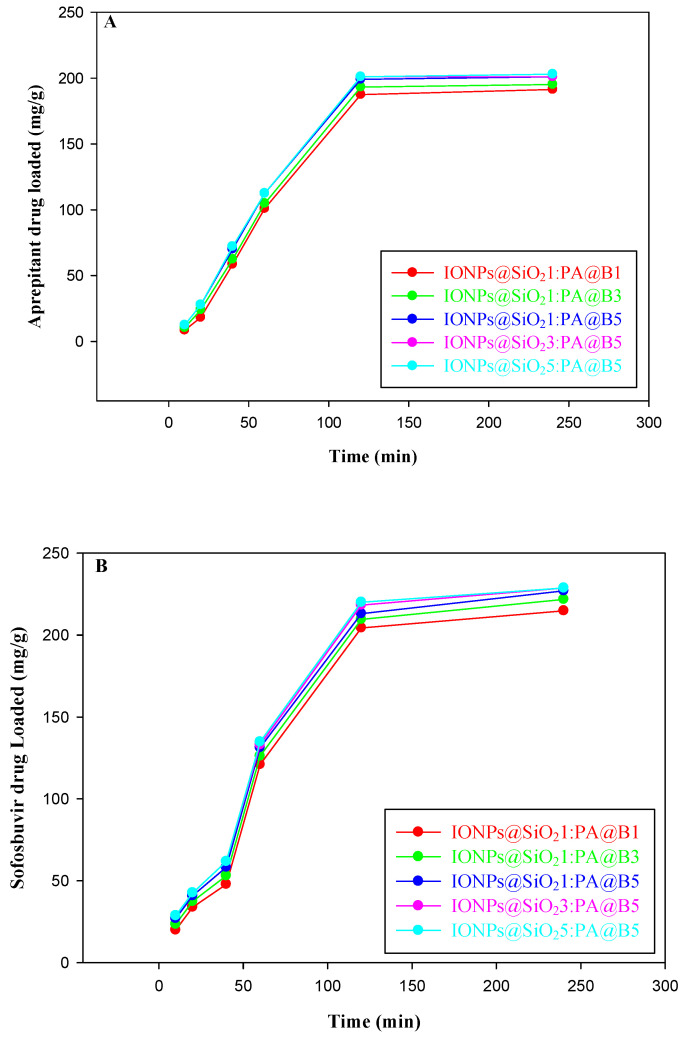
Loading of drugs into the fabricated MCM-PA@B at different time points: aprepitant (**A**); sofosbuvir (**B**).

**Figure 9:**
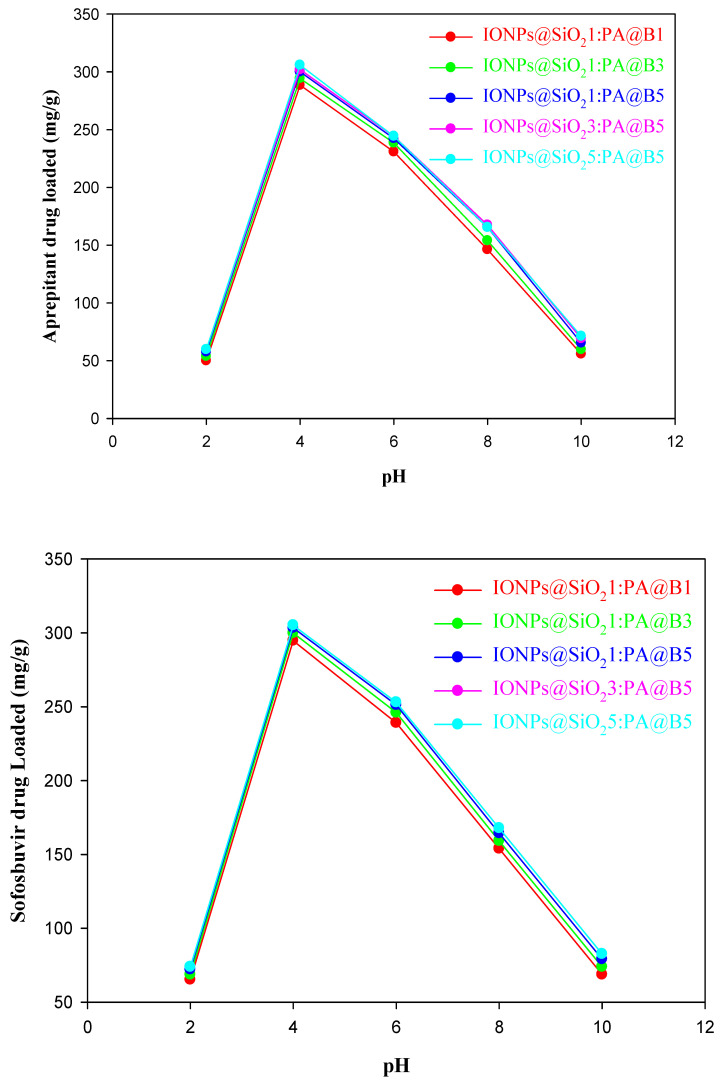
Loading of drugs into the fabricated MCM-PA@B at different pH values: aprepitant (**upper**); sofosbuvir (**lower**).

**Figure 10:**
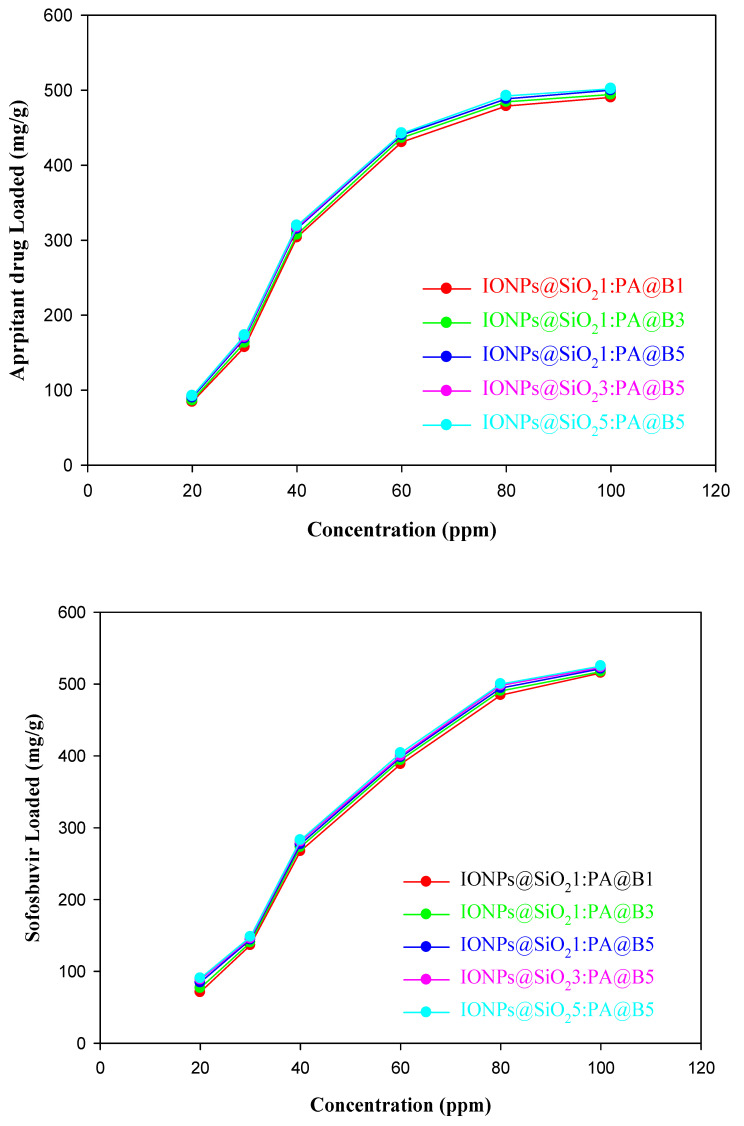
Loading of drugs into the fabricated MCM-PA@B at different concentrations: aprepitant (**upper**); sofosbuvir (**lower**).

The loading of the drugs aprepitant and sofosbuvir at various times, pH values, and concentrations is given in Figure 8, Figure 9 and Figure 10, respectively. It can be observed from the figures that the MCM-PA@B with a lower ratio of PA@B has lower loading, while it was found to be higher in PA@B in the MCM-PA@B. This might be due to the presence of higher attachment sites (functional groups) to which the drug(s) can attach, ultimately leading to higher drug loading. Moreover, as the amounts of IONPs@SiO_2_ are increased, there is no observable increase in the loading capacity of the drugs, which might be due to the incorporation of IONPs@SiO_2_ with the core–shell latex molecules. As shown in the figure, maximum loading of the drugs occurs at a quantity of 229 mg/g for aprepitant and 203 mg/g for sofosbuvir at a time of 3 h. After 3 h, there was no increase in the loading capacity of the drug, showing that all of the attachment sites were occupied and, hence, equilibration had occurred.

As previously shown in Figure 4, the synthesized composite materials have a pHzpc of 2.5. Above this pH, MCM-PA@B has positive charge, while below this pH it has negative charge. According to Bedi et al. [49], aprepitant has a pKa value of 9.7, while Sofosbuvir has a pKa value of 9.3 [50]. These pKa values show that both drugs are in an anionic form below pH 9. Maximum loading of the drugs was found to be 307 mg/g for aprepitant and 306 mg/g for sofosbuvir at pH 4 due to the presence of opposite charges on the drugs and composite materials. The loading capacity decreases as the pH is increased from 4 to 10, due to conversion of the drugs to a cationic form, which causes repulsion at higher pH. The lesser amount of drug loading is attributed to the free space available for the accommodation of drug molecules.

The loading of both drugs was also studied for different concentrations of the drugs. From Figure 10 (upper), it can be observed that the loading of aprepitant reached a maximum of 490 mg/g for composite materials with a lower quantity of core–shells and 501 mg/g for composites with higher quantities of PA@B. The increase in the loading of the drug occurred until 80 ppm, and then there was no significant change in the loading amount of the drug. The loading of sofosbuvir increased to maximum values of 515 mg/g for composite materials with lower quantities of core–shells and 525 mg/g for composites with higher quantities of core–shell latex. The loading of sofosbuvir into the composite materials is given in Figure 10 (lower).

**Figure 11:**
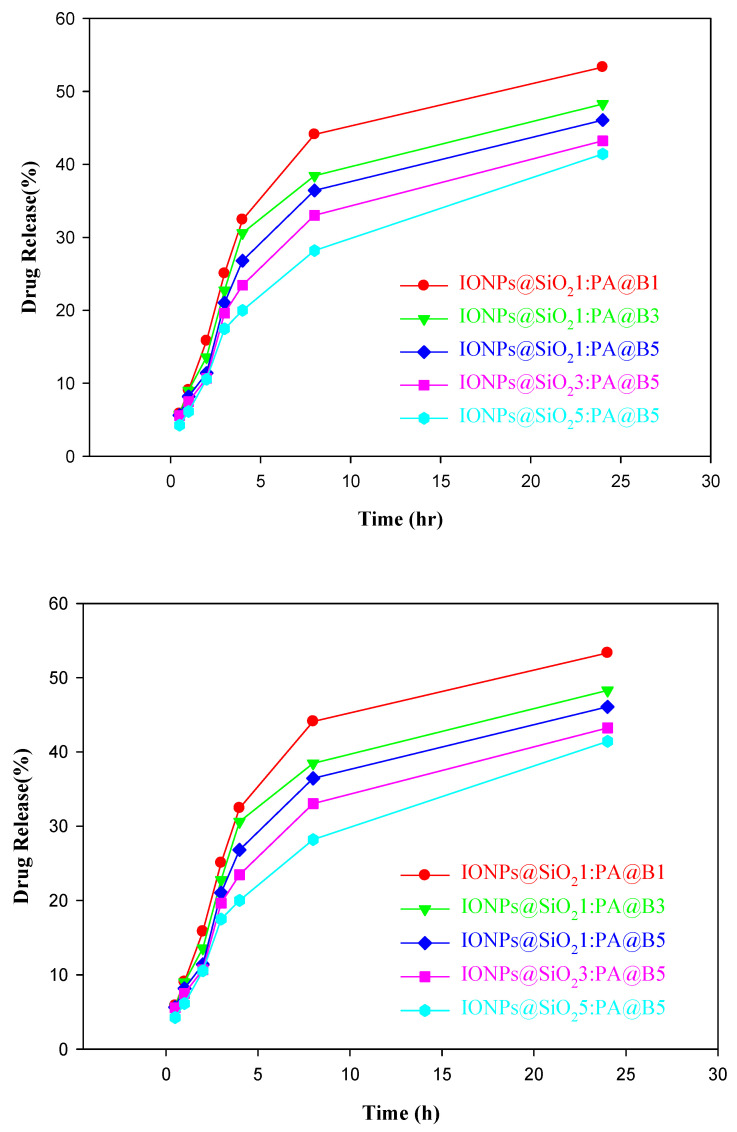
Controlled release study of drugs from the fabricated MCM-PA@B: aprepitant (**upper**); sofosbuvir (**lower**).

The release of the drugs aprepitant and sofosbuvir at various time intervals is shown in Figure 11.

### 3.8. Adsorption Kinetics Models

Various adsorption kinetics models were applied tothe loading of both drugs onto the fabricated MCM-PA@B. Table 2A,B show the data of the kinetic model applied. As we know that first-order kinetics is concentration-dependent, first-order kinetics explains that the incorporation of drugs into the MCM-PA@B is directly dependent on the amount of the drug incorporated and its saturation concentration. From the table, we can see that R^2^ has maximum values of 0.92 and 0.94 for MCM-PA@B, informing us that this first-order kinetic model is not enough to explain the loading of both drugs.

The second-order kinetics equation is dependent on the loading equilibrium capacity, which assumes that the rate of loading over the sites of attachment is proportional to the square of unavailable sites for attachment. The rate of loading is related to the concentration of the activated sites on the surface of MCM-PA@B. According to Table 2A,B, second-order kinetics for loading of both drugs fits well due to its higher R^2^ value of 1. This high R^2^ value shows that in the loading of both of the drugs, the rate-controlling step could be chemisorption. The formation of a link between the hydrogel and the drug takes place through exchange of electrons, which is chemisorption [51].

The diffusion process plays a very crucial role in the loading of drugs into the composite materials. According to Table 2A,B, the obtained data show good correlation for the second-order kinetics model, but cannot explain the drug diffusion mechanism. The values obtained initially for the loading of drugs can be used to evaluate the intra-particle diffusion model of kinetics. Inside the active sites of MCM-PA@B, the loading of both of the drugs may be controlled by mass transfer through pores, liquid-phase external mass transfer, or both [52]. The rate-determining step of the loading of drugs may be pore diffusion or film diffusion. However, we can conclude from the data in Table 2A,B that the intra-particle diffusion step was not involved in the loading of either drug. The results of the intra-particle diffusion models shows that three steps are involved in the loading of drugs: In the initial step, drug molecules are loaded at quicker rate from the solution, with active sites of composite materials on their surface [53]. During the second stage, the drug molecules go inside the space present in composite materials at a quicker rate. In the third stage, the drug molecules diffuse inside the composite materials through very tiny pores—where the diffusion takes place very slowly—and reach their maximum quantity, after which no further loading takes place. From all of these results obtained for the loading of both of the drugs we can conclude that this kinetic model is not the rate-controlling step for the loading of both of the drugs, for which this model does not pass through the origin.

### 3.9. Application of Equilibrium Isotherms on the Loading of Drugs

Langmuir and Freundlich equilibrium isotherms were used to evaluate the loading of drugs into the fabricated composite materials. The data for equilibrium isotherms are given in Table 3A,B. The interaction between the drugs and composite materials was evaluated through these isotherms. The loading of drugs over the uniform surface of the composite materials through monolayer incorporation is shown by the Langmuir adsorption isotherm. Both of the drugs follow Langmuir isotherms, for which the R^2^ value is 1, showing us that the loading of both drugs is favorable, due to the presence of many numbers of active sites as well as the uniform surface of the composite materials. The data for the Freundlich isotherms for both of the drugs show that the loading of the drugs does not follow them satisfactorily, with n values of less than 1 as compared to the Langmuir isotherms [54].

## 4. Conclusions

In this study, IONPs@SiO_2_ and CS were successfully synthesized and incorporated with one another to produce composite materials. The synthesis of these composite materials was confirmed by the FTIR, TGA, XRD, and SEM analysis techniques. From the TGA, it was found that the composite materials with high ratios of IONPs@SiO_2_ were more thermally stable. All of the synthesized composite materials were further applied for the loading and release of aprepitant and sofosbuvir. Maximum loading was found at pH 4 and at a time of 2 h; moreover, this loading amount increased with the increase in the concentration of the drugs. These loaded composite materials were further investigated for their release at pH 7.4 and 37 °C. The release of the drugs reached a maximum of 50% after 24 h. From all of the data obtained, we can conclude that increasing the amount of CS and IONPs@SiO_2_ in the composite materials can enhance the loading efficiency of both drugs, while slow release of the drugs occurs in these composite materials. All of our results show that the composite materials of IONPs@SiO_2_ with CS can be used as efficient drug delivery systems.

## Figures and Tables

**Table 1:** Different ratios of IONPs@SiO_2_ and PA@B in the synthesis of MCM-PA@B.

S. No.	IONPs@SiO_2_:PA@B	IONPs@SiO_2_ (g)	PA@B (mL)	ABCA (mL)
1	IONPs@SiO_2_1:PA@B1	0.1	1	2
2	IONPs@SiO_2_1:PA@B3	0.1	3	2
3	IONPs@SiO_2_1:PA@B5	0.1	5	2
4	IONPs@SiO_2_3:PA@B5	0.3	5	2
5	IONPs@SiO_2_5:PA@B5	0.5	5	2

**Table 2:** (**A**). Kinetic models for loading of aprepitant into the fabricated IONPs@SiO_2_-PA@B composite materials. (**B**). Kinetic models for the loading of sofosbuvir into the IONPs@SiO_2_-PA@B composite materials.

(**A**)					
	**IONPs@SiO_2_1:PA@B1**	**IONPs@SiO_2_1: PA@B3**	**IONPs@SiO_2_1: PA@B5**	**IONPs@SiO_2_3: PA@B5**	**IONPs@SiO_2_5: PA@B5**
qe (exp) mg/g	193.82	193.903	194.02	194.02	194.06
**Pseudo-first-order kinetic model**					
K_1_(min^−1^)	−0.035	−0.18	−0.042	−0.015	−0.042
qe (mg/g)	8.18	9.79	9.86	4.56	9.98
R^2^	0.92	0.91	0.91	0.92	0.91
**Pseudo-second-order kinetic model**					
K_2_(min^−1^)	0.001	0.001	0.001	0.001	0.001
qe (mg/g)	197.08	197.08	197.08	197.08	197.08
R^2^	1	1	1	1	1
**Intra-particle diffusion model**					
K_int_	0.336	0.338	0.342	0.342	0.345
C	189.18	189.25	189.33	189.34	189.32
R^2^	0.901	0.898	0.897	0.893	0.898
(**B**)					
	**IONPs@SiO_2_1:PA@B1**	**IONPs@SiO_2_1:PA@B3**	**IONPs@SiO_2_1:PA@B5**	**IONPs@SiO_2_3:PA@B5**	**IONPs@SiO_2_5:PA@B5**
qe (exp) mg/g	194.29	194.43	194.53	194.57	194.57
**Pseudo-first-order kinetic model**					
K_1_(min^−1^)	−0.027	−0.026	−0.025	−0.027	−0.029
qe (mg/g)	7.05	6.92	6.73	7.24	7.57
R^2^	0.93	0.94	0.94	0.93	0.93
**Pseudo-second-order kinetic model**					
K_2_(min^−1^)	0.001	0.001	0.001	0.001	0.001
qe (mg/g)	196.07	196.07	196.07	196.07	196.07
R^2^	1	1	1	1	1
**Intra-particle diffusion model**					
K_int_	0.366	0.366	0.366	0.366	0.370
C	187.37	187.37	189.37	189.41	189.42
R^2^	0.895	0.899	0.902	0.898	0.893

**Table 3:** (**A**). Equilibrium isotherms and their values for the loading of aprepitant on the IONPs@SiO_2_-PA@B composite materials. (**B**). Equilibrium isotherms and their values for the loading of sofosbuvir on the IONPs@SiO_2_-PA@B composite materials.

(**A**)					
	**IONPs@SiO_2_1:PA@B1**	**IONPs@SiO_2_1:PA@B3**	**IONPs@SiO_2_1:PA@B5**	**IONPs@SiO_2_3:PA@B5**	**IONPs@SiO_2_5:PA@B5**
N	−24.69	−25	−25.31	−25.31	−25.38
K_f_	216.57	216.27	215.97	216.02	215.87
R^2^	0.99	0.99	0.99	0.99	0.99
K_L_ (L/g)	−243.9	−250	−256.41	−256.41	−256.41
A_L_ (L/mol)	−1.317	−1.35	−1.38	−1.38	−1.38
Qͦ	185.18	185.18	185.18	185.18	185.18
R^2^	1	1	1	1	1
(**B**)					
	**IONPs@SiO_2_1:PA@B1**	**IONPs@SiO_2_1:PA@B3**	**IONPs@SiO_2_1:PA@B5**	**IONPs@SiO_2_3:PA@B5**	**IONPs@SiO_2_5:PA@B5**
N	−25.83	−26.24	−26.52	−26.73	−26.80
K_f_	215.42	215.07	214.78	214.58	214.53
R^2^	0.99	0.99	0.99	0.99	0.99
K_L_ (L/g)	−270.27	−277.77	−277.77	−285.71	−285.71
A_L_ (L/mol)	−1.45	−1.5	−1.5	−1.54	−1.54
Qͦ	185.18	185.18	185.18	185.18	185.18
R^2^	1	1	1	1	1
